# Pneumococcal Otitis Media by Contagion

**Published:** 1909-05-08

**Authors:** 


### Otology.

### PNEUMOCOCCAL OTITIS MEDIA BY CONTAGION.

That lobar pneumonia is contagious under certain circumstances is generally allowed. It would doubtless be very difficult to prove statistically that the transmission of pneumonia by contact is more than coincidence; clinically, however, the impression that actual contagion occurs is sometimes brought home to one very strongly.

The pneumococcus is present in normal throats, of course, and it requires either some great increase in the virulence of the micro-organism, or considerable diminution in the patient's general powers of resistance, for a pathological result to ensue. In many cases of apparent contagion it may really be exhaustion of the patient that has led to the victory of the patient's own pneumococci rather than of pneumococci caught from another person. Be this as it may, the possibility of pneumococcal infections being conveyed from one person to another must be allowed; and seeing that pneumococci can produce many different lesions besides pneumonia, it is not surprising if lobar pneumonia in one person may cause acute otitis media in another, as seems to have happened in the following case:

A diabetic patient, aged 65, was attacked by lobar pneumonia at the right base on June 24. There were crepitant râles and bronchial breathing in the right axillary region, and the sputum was viscid and rusty. Notwithstanding the patient's age and the associated glycosuria, recovery took place, the crisis occurring upon the ninth day and convalescence being natural. The patient's wife attended him night and day from the beginning, seeing to the sputum vessels, sponging him and making his bed herself. Perfectly well hitherto, and without any previous illness to be noted, she began to suffer on June 27, three days after her husband's pneumonia set in, from purulent discharge from both ears. The pus had no appreciable smell, and was whitish in colour, opaque, and very slightly tinged with blood. There was very little constitutional disturbance, and under simple antiseptic treatment she rapidly got well, the discharge being almost *nil* by the twelfth day. Perforation of each membrana tympani was seen at first, but cicatrisation speedily occurred.

The development of this double otitis media, acutely and quite independently of any former illness, in a woman who had been closely associated with a case of lobar pneumonia for three days, raised the question of whether or not it was an instance of contagion. The pus coming from the ear was investigated at a bacteriological laboratory, and pneumococci were found in abundance both in films of the pus and in cultures from it. The case was clearly one of pneumococcal otitis media.

It seems likely that the pneumococci could have been conveyed from the husband to the wife either in the air she breathed or by her hands becoming soiled in dealing with the sputum. According to Zaufal, the diplococcus pneumoniæ would seem to be the ordinary pathogenic agent in acute otitis media arising without apparent cause, or as the result of a chill; whereas pyogenic micro-organisms are most often found in secondary otitis media. In this case the pneumococcal lesions may have arisen coincidently in husband and wife, but the possibility of the one having caught it from the other cannot be gainsaid, so that it would be quite wise to recommend that nurses and other persons who may happen to be closely associated with a case of pneumonia should observe similar precautions to those adopted in cases of phthisis, both as regards the treatment of the patient's sputum, and as regards being themselves out of doors in the fresh air as much as possible when off duty. Antiseptic measures for the hands would not be out of place either.

